# Exploring teachers’ perspectives on integrating theory and practice in secondary school physical education

**DOI:** 10.3389/fspor.2026.1932526

**Published:** 2026-10-29

**Authors:** Siti Nursyahiirah Hasan, Annisaa Basar, Syed Kamaruzaman Syed Ali, Siti Nursyafiqah Hasan

**Affiliations:** Department of Educational Foundations and Humanities, Faculty of Education, Universiti Malaya, Kuala Lumpur, Malaysia

**Keywords:** basic qualitative inquiry, physical education, secondary school, teacher perspectives, theory–practice integration

## Abstract

**Introduction:**

The integration of theoretical knowledge and practical teaching remains an ongoing pedagogical challenge in school-based Physical Education. This study explored teachers' perspectives on integrating theory and practice in secondary school Physical Education.

**Methods:**

A Basic Qualitative Inquiry design was employed, involving semi-structured interviews with ten Physical Education teachers selected through purposive sampling. Data were analysed through manual coding and thematic analysis to identify recurring pedagogical patterns.

**Results:**

The findings indicated that theory–practice integration was commonly implemented sequentially or through informal instructional practices rather than structured lesson planning. Structural constraints, particularly limited instructional time and large class sizes, together with professional constraints related to the lack of structured teaching modules and variation in teacher competence, influenced teachers' instructional decisions. While practical instruction enhanced student engagement and motivation, insufficient theoretical integration limited students' conceptual understanding. The findings informed an inductively derived conceptual model explaining how structural and professional constraints shape theory–practice integration and student learning experiences.

**Discussion:**

The study highlights the need for clearer pedagogical frameworks, structured teaching resources, and professional development to support effective theory–practice integration in secondary school Physical Education.

## 1 Introduction

Physical Education (PE) is an essential component of formal education that plays a significant role in developing students’ overall potential, encompassing physical, cognitive, and affective dimensions. In contemporary educational settings, PE is no longer viewed merely as a context for physical activity but rather as a pedagogical medium that supports meaningful learning through the integration of theoretical knowledge and practical application. Within modern pedagogical perspectives, effective PE learning requires a balance between conceptual understanding and the performance of physical skills so that students can connect knowledge with authentic learning experiences. Consequently, the integration of theory and practice is increasingly recognised as an important element in enhancing the quality of PE teaching in schools.

Recent literature indicates that effective sport instruction requires structured pedagogical approaches that integrate conceptual understanding with practical performance (1, 2) consistent with established Physical Education pedagogy emphasising meaningful, structured learning experiences (3, 4). These models and their pedagogical implications are discussed in detail in the Literature Review (Sections 2.1 and 2.2).

Nevertheless, the implementation of sport instruction in PE often faces challenges in maintaining a balance between theoretical instruction and practical activities. Previous research indicates that although efforts have been made to strengthen teachers’ competencies and improve the quality of PE instruction (5, 6), issues related to pedagogical integration remain insufficiently explored. As a result, the ways in which theoretical and practical components are integrated in everyday Physical Education teaching remain insufficiently understood. Evaluations of teachers’ competencies in applying fitness monitoring and physical literacy approaches demonstrate that pedagogical elements play a critical role in determining learning effectiveness (5). Similarly, the development of instruments to assess PE teaching quality emphasises that teachers’ perceptions of students’ learning experiences are influenced by the instructional structures and teaching approaches employed (6).

In the Malaysian context, the importance of PE and sports in student development has long been recognised within the national education system. However, variations in students’ physical activity and physical fitness highlight the continuing importance of promoting meaningful participation in physical activity within educational settings (7). Research examining PE teachers’ experiences in challenging teaching situations also suggests that pedagogical adaptation and clarity in instructional structure significantly influence learning effectiveness (8). In addition, teachers’ confidence in managing instruction has been shown to affect the teaching approaches adopted in the classroom (9). These findings indicate that teachers’ pedagogical competence is a fundamental factor in determining the effectiveness of PE instruction.

Despite these developments, a significant gap remains in the integration of theory and practice in sport instruction at the secondary school level. In particular, there is a lack of structured integration between theoretical instruction and the implementation of sport skills, where theoretical elements such as movement principles, game strategies, and safety considerations are not systematically integrated with practical activities. In many situations, PE teaching tends to emphasise physical activity without reflective discussion of the underlying concepts. This situation raises concerns that without a strong pedagogical foundation, PE may risk being perceived as a less academic subject (10).

Furthermore, the level of emphasis placed on practical sport-based learning is often not optimally implemented. Although physical activities are conducted during PE classes, they are not necessarily designed to develop sport skills progressively and meaningfully. Research on the development of comprehensive sport training modules suggests that systematic instructional structures can significantly enhance skill acquisition (11). Similarly, studies examining structured and unstructured physical activities indicate that structured approaches produce more consistent outcomes in the development of students’ motor skills (12). These findings suggest that the lack of structure in practical instruction may negatively affect the quality of students’ learning.

In addition, research in sport pedagogy highlights that teacher autonomy and motivational support play important roles in enhancing student engagement in learning (13). When teaching is meaningfully designed and supported by clear pedagogical approaches, students demonstrate higher levels of engagement and self-efficacy. Therefore, the integration of theory and practice is not only related to instructional content but is also closely associated with students’ learning experiences and motivation.

Based on this background, this study aims to explore the pedagogical significance of integrating theoretical and practical sport instruction in the context of secondary school Physical Education in Malaysia. Specifically, this study seeks to identify the factors contributing to the lack of structured integration between theoretical instruction and practical sport teaching in secondary school Physical Education. In addition, the study examines teachers’ perceptions of how the level of emphasis on practical instruction influences students’ learning experiences.

In terms of significance and contribution, this study provides important implications for educational policymakers by offering empirical evidence on the need to strengthen pedagogical guidelines for PE. For governments and stakeholders, the findings may serve as a basis for improving curriculum implementation, teacher training, and the monitoring of PE practices in secondary schools. From a community perspective, more structured and meaningful sport instruction has the potential to cultivate a generation that is more physically active and health-conscious. The education and sports industries may also benefit from the findings through the development of training modules, instructional materials, and innovative pedagogical approaches. From a scholarly perspective, this study contributes to the literature on Physical Education pedagogy by emphasising the integration of theory and practice within the Malaysian context, which remains relatively underexplored in previous research.

The scope of this study focuses on Physical Education teachers in Malaysian secondary schools as the primary participants. The study aims to develop a deeper understanding of how teachers plan and implement the integration of theory and practice in PE instruction and how these approaches influence students’ learning experiences. Although students were not directly involved as participants, students’ learning experiences were analysed based on teachers’ perceptions and reflections on their teaching practices. Therefore, this study adopts a qualitative approach to explore teachers’ perspectives on the integration of theoretical and practical instruction in Malaysian secondary school Physical Education.

## 2 Literature review

### 2.1 Theory-practice integration in physical education

The integration of theoretical instruction and practical application in Physical Education (PE) has increasingly attracted attention in educational literature, as it is considered fundamental to meaningful and effective learning. Conceptually, PE is not merely a platform for physical activity but a pedagogical process that combines conceptual understanding, motor skill development, and students’ social development. Contemporary pedagogical models such as the Sport Education Model and the Tactical Games Model emphasise the importance of integrating theoretical and practical elements in sport instruction (14). These approaches suggest that students develop a deeper understanding of game strategies and movement principles when theoretical explanations are contextualised through practical activities. However, systematic research indicates that the implementation of such pedagogical models is not always consistent in actual teaching practice (2), found that sport instruction in secondary schools is often activity-oriented without a clearly structured pedagogical framework. This suggests that structured integration between theoretical and practical components may be lacking in many teaching contexts (15). Similarly demonstrated that systematically designed teaching programmes contributed to the development of sport-specific skills among secondary school students, reinforcing the importance of pedagogical structure in supporting effective learning.

### 2.2 Pedagogical models and instructional structure in sport education

Pedagogical models such as the Sport Education Model emphasise structured learning environments that integrate skill development, tactical understanding, and student participation in authentic sport contexts (4). These models aim to promote meaningful learning experiences and long-term engagement in physical activity (3). Curriculum design and instructional planning also influence the extent to which theoretical and practical components are integrated (16, 17).

### 2.3 The role of teacher self-efficacy and professional competence

Teacher self-efficacy has been found to have a direct relationship with the instructional approaches adopted in the classroom (18, 19). Collectively, these findings suggest that internal teacher-related factors may contribute to the lack of structured integration if pedagogical confidence and competence are insufficient.

### 2.4 Student engagement and learning experiences

Teacher support has been shown to increase students’ autonomous motivation and self-efficacy in PE contexts (20, 21). These findings reinforce the argument that the quality and emphasis of practical sport instruction can shape students’ learning experiences in PE.

### 2.5 Research gap and the need for the present study

Despite the extensive literature on pedagogical models, teacher self-efficacy, and student engagement, a significant gap remains regarding structured integration between theoretical and practical instruction in secondary school Physical Education, particularly within the Malaysian context. Therefore, the present study aims to explore teachers’ perspectives on theory–practice integration and to examine how the level of emphasis on practical instruction influences students’ learning experiences.

### 2.6 Synthesis: toward an empirical understanding of theory-practice integration

Taken together, the literature reviewed above suggests that theory-practice integration in Physical Education is shaped by the interaction of at least three interrelated strands. First, pedagogical models such as the Sport Education Model and the Tactical Games Model articulate what structured integration should look like, yet their implementation is not always consistent in everyday teaching (2, 14). Second, teacher-related factors, particularly self-efficacy, professional training, and content knowledge, condition teachers’ capacity to enact these models in practice (18, 19, 22). Third, the degree to which integration is achieved has downstream consequences for students’ engagement, motivation, and conceptual understanding (20, 23).

However, most existing studies examine these strands separately, either evaluating a pedagogical model's effectiveness or the correlates of teacher self-efficacy or student engagement in isolation. Comparatively little is known about how these three strands interact in teachers’ actual day-to-day instructional decisions, particularly within the Malaysian secondary school context. This gap motivates the present study's exploratory, teacher-perspective approach. Rather than testing an existing pedagogical model, this study inductively examines how teachers themselves describe and negotiate theory-practice integration; the empirical patterns that emerge from this analysis are subsequently synthesised into a conceptual model in Section 4.1, following the Findings, consistent with the interpretive logic of Basic Qualitative Inquiry.

## 3 Methodology

This study employed a qualitative approach using a Basic Qualitative Inquiry design to explore teachers’ perspectives on the integration of theoretical and practical instruction in Physical Education (PE) in Malaysian secondary schools. A qualitative approach was selected because the study aimed to understand teachers’ experiences, interpretations, and meanings constructed within the context of their actual teaching practices (24, 25).

### 3.1 Participants

A total of ten secondary school Physical Education teachers participated in this study. Participants were selected through purposive sampling to ensure that they had direct experience in teaching sport skills and were actively involved in planning PE lessons (26). The demographic characteristics of the ten participating Physical Education teachers are presented in Table 1.

**Table 1:** Demographic characteristics of the participants.

Participant	Gender	Teaching experience (years)	Teaching subject	School type
P1	Female	6	Physical Education	Secondary School
P2	Female	11	Physical and Health Education	Secondary School
P3	Male	21	Physical Education	Secondary School
P4	Female	17	Physical Education	Secondary School
P5	Male	13	Physical Education	Secondary School
P6	Male	11	Physical and Health Education	Secondary School
P7	Female	10	Physical and Health Education	Secondary School
P8	Male	25	Physical Education	Secondary School
P9	Female	13	Physical Education	Secondary School
P10	Female	23	Physical Education	Secondary School

### 3.2 Data collection

The primary method of data collection was semi-structured interviews. This approach allowed participants to elaborate on their experiences and perspectives in an open and reflective manner (27). The interview questions were developed in alignment with the problem statements and research objectives, focusing on the planning of theory-practice integration, factors constraining such integration, and teachers’ perceptions of how practical instruction influences students’ learning experiences. In addition to the interviews, limited non-participant observations were conducted with the same ten participants to provide contextual support for the interpretation of the interview data. These observations were not treated as an independent data source for thematic analysis.

### 3.3 Data analysis

All interviews were audio-recorded and fully transcribed prior to analysis. The interview transcripts were analysed through an iterative process of coding and thematic analysis to identify patterns and emerging themes across the participants’ narratives, involving repeated readings of the transcripts, the identification of initial codes, the grouping of codes into categories, and the development of broader themes (24). Data coding was conducted by the primary researcher responsible for data curation and formal analysis. The coded transcripts were subsequently reviewed by the co-authors as part of the validation process indicated in the Credit Author Statement, and any discrepancies in interpretation were resolved through discussion among the research team. Reflexive memos were also maintained throughout data analysis to document analytical decisions and reduce potential subjectivity.

Data collection and analysis proceeded iteratively, with participants’ accounts compared across successive interviews to identify recurring and newly emerging codes and themes. Saturation was considered to have been reached when the analysis of subsequent interviews did not generate substantively new codes, categories, or themes relevant to the research objectives. Data collection was concluded after ten participants when the existing thematic structure was sufficiently developed and additional accounts primarily reinforced patterns already identified. Consistent with recommendations for purposive sampling in qualitative research, the sample was considered adequate to capture a sufficient range of perspectives relevant to the study's objectives and to support depth of understanding rather than statistical generalisation (24, 26).

### 3.4 Ethical considerations

Ethical approval for this study was obtained from University of Malaya Research Ethics Committee (UMREC) [Reference No. UM.TNC(P&I)/UMREC/3/6056]. Written informed consent was obtained from all participants prior to the interviews. Participants’ identities were protected through the use of pseudonyms or coded identifiers, and all data were securely stored for academic purposes only (25).

### 3.5 Trustworthiness

To ensure the credibility and trustworthiness of the findings, member checking and an audit trail were maintained. Member checking was conducted through face-to-face sessions with all ten participants, in which the preliminary findings and emerging themes were shared and discussed to verify that the interpretations accurately reflected participants’ experiences. Participants’ feedback was consistent with the researchers’ interpretation of the data, which supported the credibility of the identified themes. An audit trail was maintained through systematic documentation of the coding and analytical process, tracing the development of codes and themes from the original transcripts, which was available for review throughout the analysis process.

## 4 Findings

The thematic analysis of interview data revealed three main themes related to the integration of theoretical and practical instruction in Physical Education (PE): (1) Sequential and Informal Implementation of Theory–Practice Integration, (2) Structural and Professional Constraints in Physical Education Teaching, and (3) Student Learning Experiences. These themes explain how teachers implement and experience theory-practice integration in secondary school Physical Education. The thematic structure and its associated subthemes are illustrated in Figure 1, while the coding structure and representative supporting quotations are presented in Table 2.

**Figure 1:**
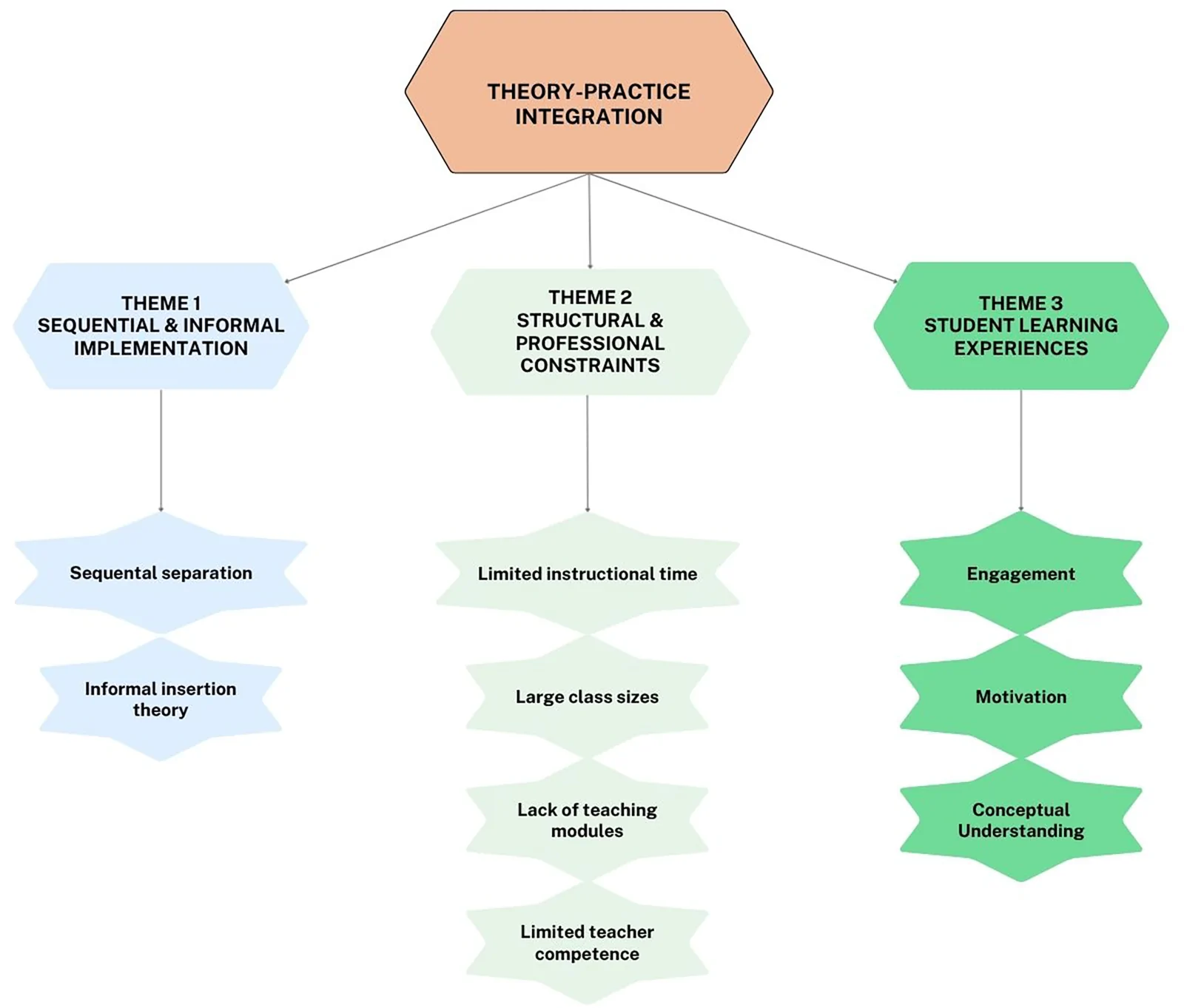
Thematic structure of theory-practice integration in secondary school physical education.

**Table 2:** Coding structure and supporting evidence for the identified themes.

Main theme	Coding/subtheme	Supporting quote
Sequential and Informal Implementation of Theory–Practice Integration	Sequential separation between theory and practice	“The first week is theory… the second week is practical.” (P1) “In the first week I explain the topic in the classroom…the second week I conduct the practical session because time is limited”
	Informal insertion of theory during practical activities	“While they were playing, I threw in a little theory…” (P8)
Structural and Professional Constraints in Physical Education Teaching	Time constraint and limited instructional time	“Many students, short time.” (P2) “There are many students and the time is short…if we try to cover both, the students might end up waiting too long.”
	Lack of professional training or teaching modules	“There is no special module…” (P6) “There is no specific module to systematically combine theory and practical instruction.”
Student Learning Experiences	Increased student motivation and participation	“Students become enthusiastic.” (P1) “If we teach with knowledge and experience, the students become more enthusiastic…they want to participate, not just watch.”
	Improved conceptual understanding through theory–practice integration	“When theory and practice go hand in hand, students understand.” (P3)

Figure 1 presents the thematic structure derived from the thematic analysis. Three overarching themes emerged from the interview data, namely Sequential and Informal Implementation, Structural and Professional Constraints, and Student Learning Experiences. Together, these themes and their associated subthemes provide the analytical foundation for the inductively derived conceptual model presented in Figure 2. The coding structure and representative supporting quotations for each subtheme are presented in Table 2.

**Figure 2:**
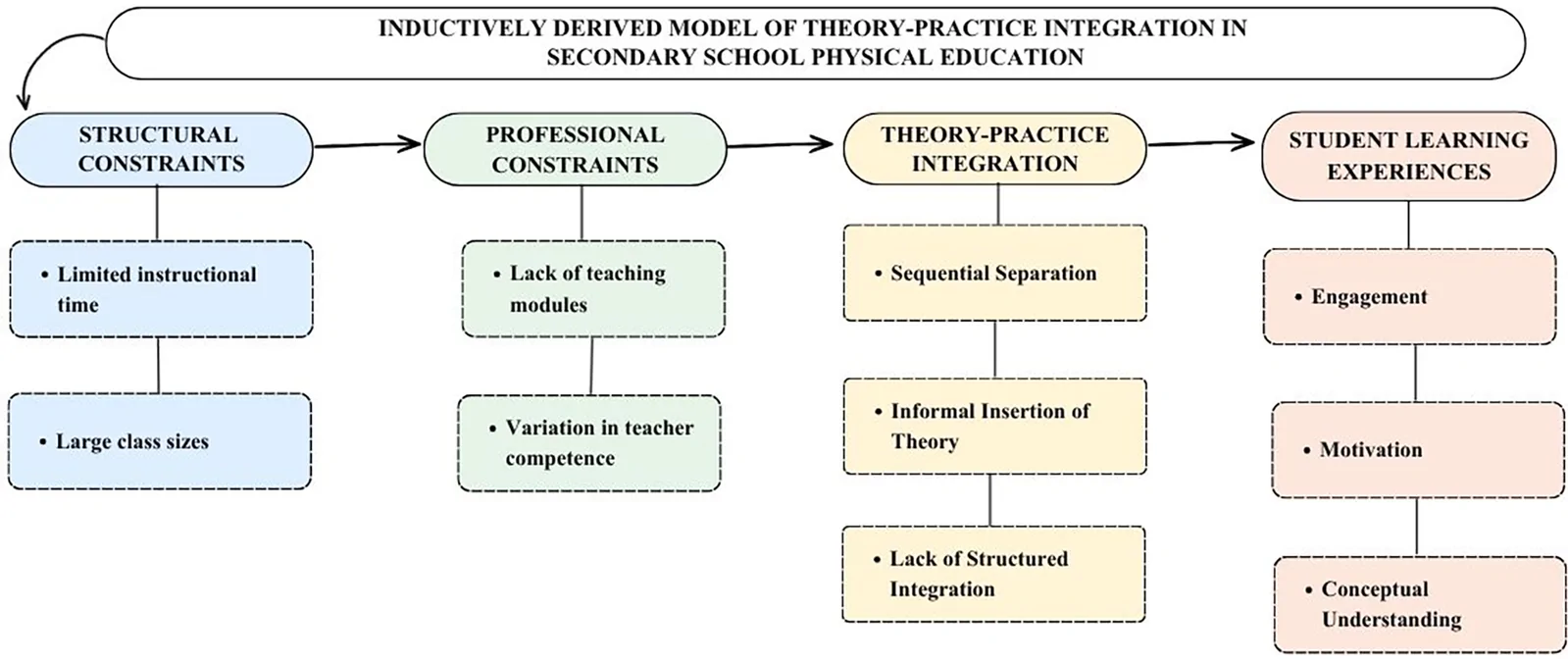
Inductively derived model of theory-practice integration in secondary school physical education. The model was developed inductively from the themes identified through thematic analysis. All constructs included in the model are directly supported by participants’ accounts presented in the Findings section.

### 4.1 An inductively derived model: structured integration of theory-practice

The three themes generated through thematic analysis were subsequently synthesised into a conceptual model termed the Structured Integration Model of Theory-Practice in Secondary School Physical Education. Consistent with the inductive logic of Basic Qualitative Inquiry, this model was not imposed on the data as a pre-existing theoretical framework; rather, it was developed after data analysis as an interpretive synthesis of the patterns identified in the Findings.

The model identifies two categories of influence that were directly evidenced in participants’ accounts: structural constraints, specifically instructional time and large class sizes, and professional constraints, specifically the absence of structured teaching modules and variation in teacher competence or confidence across different sports. These factors shaped whether theory-practice integration occurred through structured planning, informal insertion, or sequential separation, which in turn influenced student engagement, motivation and conceptual understanding, as reported by participants.

Constructs such as facilities, curriculum structure, institutional positioning, professional training pathways, and teaching experience are theoretically relevant to theory-practice integration and are noted here as proposed directions for future empirical investigation, as they were not directly evidenced by participant accounts in the present dataset (and are therefore not depicted in Figures 1, 2). The relationships between structural conditions, teacher-related factors, theory–practice integration, and student learning outcomes are illustrated in Figure 2.

## 5 Discussion

The findings of this study provide important insights into how the integration of theoretical and practical instruction is implemented in secondary school Physical Education. Overall, the results suggest that although teachers recognise the pedagogical importance of integrating theory and practice, such integration is not consistently implemented in actual teaching practice. The first theme revealed that theory-practice integration is often implemented sequentially or informally rather than through structured pedagogical planning. In many cases, teachers delivered theoretical explanations in separate lessons before conducting practical activities.

While the present findings frame sequential and informal implementation primarily as a constraint-driven limitation, these practices may also be interpreted differently. Sequential delivery can provide teachers with an opportunity to introduce key concepts before students apply them in practical contexts, particularly when the content is unfamiliar or technically demanding. Furthermore, the informal insertion of theoretical explanations during practical activities may represent a form of adaptive teaching rather than an absence of pedagogical planning (28). Describe micro-adaptivity in Physical Education as teachers’ real-time adjustment of instructional tasks, explanations, and feedback in response to students’ learning needs.

Similarly (29), argue that effective Physical Education teaching requires balancing fidelity to pedagogical models with teacher agency, suggesting that contextual adaptation may contribute positively to student learning (30), further emphasise that teachers’ instructional adaptation is influenced by contextual factors, including school support, teacher self-efficacy, and classroom conditions. Collectively, these studies suggest that flexible instructional practices should be interpreted within the broader pedagogical and contextual environment rather than being viewed solely as indicators of ineffective teaching.

However, unlike the contexts described in these studies, the present findings indicate that teachers’ adaptations were primarily driven by contextual constraints, particularly limited instructional time, large class sizes, and the absence of structured teaching modules, rather than by deliberate pedagogical design. This distinction suggests that the sequential and informal approaches observed in the present study functioned primarily as compensatory responses to contextual limitations rather than as intentionally designed pedagogical strategies. Therefore, the effectiveness of theory-practice integration may depend not only on the instructional approach adopted but also on the contextual conditions that enable teachers to implement it effectively. These alternative perspectives indicate that sequential and informal integration should not automatically be regarded as pedagogically deficient. Accordingly, future research should distinguish between intentional pedagogical adaptation and constraint-driven instructional practices in order to better understand how theory-practice integration is implemented across different educational contexts.

The second theme highlights the role of structural and professional constraints in shaping teachers’ instructional decisions. Participants frequently reported that limited instructional time and large class sizes made it difficult to integrate theoretical explanations and practical activities within the same lesson. The absence of structured teaching modules and professional training related to theory–practice integration suggests that teachers often rely on personal experience and professional judgement when planning lessons (18, 19). This finding suggests that, in the absence of structured pedagogical guidance, teachers develop individual instructional approaches based largely on their own experience and confidence. Consequently, teaching practices may vary considerably across schools, potentially resulting in inconsistencies in students’ learning experiences. This finding reinforces the importance of structured professional development and pedagogical support in enabling teachers to implement theory-practice integration more consistently across different school contexts. These findings also help explain why practical instruction tended to receive greater priority in teachers’ day-to-day decision making. Within limited lesson time and large classes, teachers appeared to prioritise opportunities for students to participate physically, while theoretical content was either separated into another lesson or incorporated briefly during practical activities. This pattern suggests that the predominance of practical instruction should not necessarily be interpreted as teachers undervaluing theoretical knowledge; rather, it may represent a pragmatic response to competing instructional demands. In the absence of structured modules that explicitly guide teachers in connecting conceptual content with practical activities, integration consequently becomes more dependent on individual professional judgement, experience, and confidence.

These patterns also resonate with evidence from diverse international contexts, suggesting that challenges in connecting pedagogical knowledge with practice are not unique to Physical Education in Malaysia. In Europe (31), emphasised the importance of moving beyond the separation of theory and practice by creating experiential pedagogies in which theoretical knowledge, bodily experience, reflection, and interaction are meaningfully intertwined. Similarly (29), in Portugal, demonstrated that the implementation of structured pedagogical approaches requires a balance between pedagogical guidance and teachers’ capacity to adapt instruction to contextual demands. In North American and other PETE contexts (32), found that practice-based pedagogies, including teaching rehearsals and repeated teaching, strengthened instructional effectiveness, content knowledge, and opportunities to connect pedagogical knowledge with practice. Evidence from Latin America further indicates that Physical Education teacher preparation may differ substantially in curricular structure and pedagogical orientation across institutions, highlighting the influence of educational context on how pedagogical knowledge is organised and enacted (33). In Australia (34), similarly demonstrated the value of embedding evidence-informed pedagogical approaches within teacher education and lesson planning to strengthen pedagogical competence. Collectively, these international findings reinforce the present study's interpretation that effective theory-practice integration depends not simply on teachers’ willingness to combine conceptual and practical instruction, but also on structured pedagogical guidance, professional competence, opportunities for reflective practice, and responsiveness to contextual conditions.

The third theme focuses on the pedagogical impact of practical instruction on students’ engagement and learning experiences. Practical sport-based instruction plays an important role in increasing students’ motivation, enthusiasm, and active participation, while theoretical explanations accompanying practical demonstrations support deeper conceptual understanding (20, 23). Nevertheless, practical instruction alone may be insufficient to achieve meaningful learning if students are not provided with opportunities to connect physical performance with underlying concepts and movement principles. This finding highlights the importance of maintaining a balanced integration of theoretical explanation and practical application in Physical Education lessons to promote both meaningful skill acquisition and deeper conceptual understanding.

Overall, these findings suggest that successful theory-practice integration depends not only on teachers’ pedagogical competence but also on the structural conditions that enable meaningful instructional planning and implementation within Physical Education classrooms.

## 6 Limitations

This study is limited by the relatively small sample size involving only ten Physical Education teachers, which may restrict the transferability of the findings to broader contexts. As a qualitative study, the research emphasises depth of understanding rather than statistical generalisation (25). In addition, the findings are primarily based on teachers’ perspectives without extensive triangulation using student data or curriculum documents. Although coded transcripts were reviewed by the co-authors as part of the validation process, coding was not conducted through fully independent parallel coding with formal inter-coder agreement statistics, which remains a methodological limitation. Furthermore, although limited non-participant observations were conducted to support the interpretation of interview data, these observations were not independently coded or analysed and are not reported as a distinct data source; the study therefore relies primarily on self-reported accounts rather than triangulated observational evidence. Future research should incorporate structured observation protocols and formal inter-coder reliability procedures to strengthen analytical rigour.

## 7 Conclusion

This study explored teachers’ perspectives on the integration of theoretical and practical instruction in secondary school Physical Education. The findings indicate that although teachers recognise the pedagogical importance of integrating theory and practice, such integration is not consistently implemented in actual teaching contexts. Structural constraints, professional constraints, and their interaction with student engagement together form the Structured Integration Model presented in Section 4.1. The study contributes empirical insights into teachers’ perspectives within the Malaysian secondary school context and highlights the need for clearer pedagogical frameworks and professional development.

## 8 Practical implications

The findings of this study offer several practical implications for Physical Education teaching and curriculum development. First, the results highlight the need for clearer pedagogical frameworks to support teachers in integrating theoretical and practical components within Physical Education lessons. Structured teaching modules or instructional guidelines could assist teachers in planning lessons that connect conceptual explanations with practical skill development more systematically.

Second, professional development programmes for Physical Education teachers should emphasise pedagogical strategies for theory–practice integration. Training opportunities that enhance teachers’ confidence and pedagogical competence in sport instruction may enable teachers to implement more balanced and meaningful learning experiences for students.

Third, school administrators and policymakers should consider structural factors such as instructional time allocation, class size, and access to teaching resources when designing Physical Education programmes. Addressing these structural constraints may help create more supportive environments for implementing integrated Physical Education instruction.

Finally, the findings also suggest that collaborative learning strategies, including peer-assisted learning, may provide additional opportunities for enhancing students’ engagement and skill development in Physical Education classes.

## Data Availability

The datasets presented in this article are not readily available because the datasets generated and analyzed during the current study are not publicly available due to ethical and confidentiality restrictions and the conditions of participants’ informed consent. Requests regarding the study may be directed to the corresponding author; however, access to the raw data is subject to ethical approval and institutional requirements. Requests to access the datasets should be directed to Dr. Annisaa Basar, annisaa@um.edu.my.
